# Noninvasive mechanical ventilation with average volume assured pressure support (AVAPS) in patients with chronic obstructive pulmonary disease and hypercapnic encephalopathy

**DOI:** 10.1186/1471-2466-13-12

**Published:** 2013-03-12

**Authors:** Killen Harold Briones Claudett, Monica Briones Claudett, Miguel Chung Sang Wong, Alberto Nuques Martinez, Ricardo Soto Espinoza, Mayra Montalvo, Antonio Esquinas Rodriguez, Gumersindo Gonzalez Diaz, Michelle Grunauer Andrade

**Affiliations:** 1 Pulmonology Department, Military Hospital, Guayaquil, Ecuador; 2 Department of Respiratory Medicine, Panamericana Clinic, Guayaquil, Ecuador; 3 Department of Respiratory Medicine – Intensive Care, Santa Maria Clinic, Guayaquil, Ecuador; 4 Department of Pneumology – Intensive Care, Regional Hospital of Guayaquil, Guayaquil, Ecuador; 5 Intensive Care Medicine, Santa Maria Clinic, Military Hospital, Guayaquil, Ecuador; 6 Intensive Care Medicine Panamericana Clinic and Ecuadorian Institute Social Security (IESS), Guayaquil, Ecuador; 7 Universidad San Francisco de Quito, Quito, Ecuador; 8 Intensive Care Unit and Pneumology Services of the Hospital JM Morales Meseguer, Murcia, Spain

**Keywords:** BiPAP S/T with AVAPS, BiPAP S/T (spontaneous/time), Hypercapnic encephalopathy, Chronic obstructive pulmonary disease

## Abstract

**Background:**

Non-invasive mechanical ventilation (NIV) in patients with acute respiratory failure has been traditionally determined based on clinical assessment and changes in blood gases, with NIV support pressures manually adjusted by an operator. Bilevel positive airway pressure-spontaneous/timed (BiPAP S/T) with average volume assured pressure support (AVAPS) uses a fixed tidal volume that automatically adjusts to a patient’s needs. Our study assessed the use of BiPAP S/T with AVAPS in patients with chronic obstructive pulmonary disease (COPD) and hypercapnic encephalopathy as compared to BiPAP S/T alone, upon immediate arrival in the Emergency-ICU.

**Methods:**

We carried out a prospective interventional match-controlled study in Guayaquil, Ecuador. A total of 22 patients were analyzed. Eleven with COPD exacerbations and hypercapnic encephalopathy with a Glasgow Coma Scale (GCS) <10 and a pH of 7.25-7.35 were assigned to receive NIV via BiPAP S/T with AVAPS. Eleven patients were selected as paired controls for the initial group by physicians who were unfamiliar with our study, and these patients were administered BiPAP S/T. Arterial blood gases, GCS, vital signs, and ventilatory parameters were then measured and compared between the two groups.

**Results:**

We observed statistically significant differences in favor of the BiPAP S/T + AVAPS group in GCS (*P* = .00001), pCO_2_ (*P* = .03) and maximum inspiratory positive airway pressure (IPAP) (*P* = .005), among others. However, no significant differences in terms of length of stay or days on NIV were observed.

**Conclusions:**

BiPAP S/T with AVAPS facilitates rapid recovery of consciousness when compared to traditional BiPAP S/T in patients with chronic obstructive pulmonary disease and hypercapnic encephalopathy.

**Trial registration:**

Current Controlled Trials application ref is ISRCTN05135218

## Background

Noninvasive mechanical ventilation (NIV) is used in patients with acute respiratory failure for several different etiologies [1]. The heterogeneity of different patient groups leads to varying levels of success, with the best results produced in patients with infectious exacerbations of COPD and congestive heart failure [2-4]. When NIV is initiated in patients with acute respiratory failure due to infectious exacerbations of COPD, ventilatory parameters are typically determined based on clinical assessment and changes in blood gases. In this manner, NIV support pressures are manually adjusted by an operator [5].

One of the limitations of traditional NIV is altered levels of consciousness. However, under certain circumstances, especially those produced by hypercapnic conditions [6-8], traditional NIV has produced very favorable results, even in patients with hypercapnic coma [9].

Patients with infectious exacerbations of COPD have obstruction, hyperinflation, air trapping, and increased respiratory effort and central respiratory drive. In particular, blood pCO_2_ increases, which, due to its high lipid solubility, readily crosses the blood–brain barrier, provoking acidosis in the cerebrospinal fluid and cerebral interstitial tissue [8-13].

Previous studies that recommend the use of NIV in patients with altered consciousness report a rapid recovery as soon as the ventilatory strategy is established, and most recommend early intubation and suspension of treatment if consciousness does not quickly normalize [9,10]. Altered levels of consciousness can be assessed using the Glasgow Coma Scale (GCS) [14,15], the encephalopathy scoring [16], and the Kelly-Matthay Scale (KMS) [17]. Although KMS is specifically designed to assess patients with neurological disorders on NIV, it is not commonly used in the emergency unit/ICU.

Bilevel positive airway pressure-spontaneous/timed (BiPAP S/T) with average volume assured pressure support (AVAPS) allows for setting a fixed tidal volume, and the system output automatically adjusts based on variations in inspiratory pressure to ensure the predetermined target value. Its long-term benefits have been demonstrated in patients with chronic respiratory failure, obstructive sleep apnea, and alveolar hypoventilation syndrome [18-20].

We designed this study to assess the use of BiPAP S/T with AVAPS as a ventilatory strategy in patients with chronic obstructive pulmonary disease (COPD) and hypercapnic encephalopathy (GCS < 10) and to compare these results with those from patients treated with BiPAP S/T alone, upon immediate arrival in the emergency department/ICU.

## Methods

### Patients

All patients were admitted between February 2009 and September 2011, and permission was obtained from patients or their proxies if patients were unable to answer for themselves. The study was approved by the academic and ethics committee of the School of Medicine of the Universidad San Francisco de Quito. Three hospitals in Guayaquil, Ecuador participated in the study: Hospital Militar, Clinica Panamericana, and Clinica Santa Maria. A total of 22 patients were recruited for NIV and divided into two groups of 11.

### Treatment group assignments

11 patients with infectious exacerbations of COPD and hypercapnic encephalopathy with GCS < 10 were designated to receive BiPAP S/T with AVAPS.

The control group was then selected from patients in the emergency unit with infectious exacerbations of COPD and encephalopathy (GCS < 10). Patients were treated immediately and referred to us by doctors who were unaware of the study. Each patient was treated with NIV and was selected according to: APACHE II score within 4 points, age within 10 points, pH within 0.04, GCS within 2 points, and BMI within 2 points.

### Noninvasive mechanical ventilation: BiPAP S/T with AVAPS

Ventilatory parameters were initially programmed in the BiPAP S/T mode and AVAPS with an inspiratory positive airway pressure (IPAP) maximum programmed into the device of 26 cmH2O, to IPAP minimum programmed value of 12 cmH2O and an expiratory positive airway pressure (EPAP) of 6 cmH2O. The programmed tidal volume was at 8 to 12 ml/kg of IBW, and once the patient reached clinical stability and sensory, the target Vt in our patients were reprogrammed to 6–8 ml/kg/weight according to manufacturer's specifications, the decision was made by the expert physician in charge of patient case dependent, respiratory rate was 15 breaths/min, rise time set at 300–400 ms and inspiratory time was at a minimum of 0.6 s. Were given supplements O2 via an adapter circuit close to the facemask in order to maintain SaO2 above 90%. Patients were maintained on continuous NIV initially.

Maximum IPAP received delivered, exhaled tidal volume (EVT), Vmin, and leaks were monitored through the ventilator software. We used BiPAP Synchrony with AVAPS and Autotrak (*Respironics Inc., Murrysville, Pennsylvania, USA*) and a Mirage IV series facemask *(Resmed*).

### Measurements

Arterial blood gases were measured at initial values and after 1 hour, 3 hours, 12 hours and then every 24 hours during NIV; the patient was assessed by a respiratory therapist under close supervision of a physician trained in NIV. Mask use, complications, and tolerance were also assessed.

Disease severity was assessed using the APACHE II score and GCS to determine the patient's level of consciousness. Maximum Vt, maximum IPAP, EVT, Vmin, leaks, respiratory rate, heart rate, systolic blood pressure, diastolic blood pressure, and IPAP were measured upon hospitalization, after 1 hour, 3 hours, and 12 hours, and then every 24 hours during NIV.

Exclusion criteria included facial deformity, obstruction in the upper airway from surgery or trauma, alterations of the central nervous system not related to hypercapnic encephalopathy, cardiogenic pulmonary edema, pneumothorax, pulmonary thromboembolism, hemoptysis or septic shock, emergency intubation due to cardiopulmonary arrest, and hemodynamic instability with systolic pressure below 80 mmHg.

### Discontinuation of NIV

Treatment with NIV was initially used on a continuous regimen based on patient tolerance and after normalization of arterial pH > 7.35 ventilation was given in 3-hour blocks. The weaning process was initiated when clinical stability was achieved, which was defined as respiratory rate less than 24 breaths/min, a heart rate of 90 beats/min, and improved awareness and compensation from normalized pH values, with adequate SaO_2_ in ambient air and a low percentage of inspired O_2_ (3 liters). Once the patient remained stable, NIV was discontinued.

### Control group ventilation parameters: BiPAP S/T

Ventilatory parameters were initially programmed in BiPAP S/T mode. IPAP was programmed at 12 cmH2O, EPAP was programmed at 6 cmH2O. Respiratory rate was set at 15 breaths/min, rise time set at 300–400 ms, and inspiratory time was at a minimum of 0.6 s. Progressively increased levels were IPAP in increments of 2 cmH2O according to the discretion of the attending physician. Supplements were added O2 via an adapter circuit close to the facemask to maintain SaO2 above 90%. Patients were maintained on continuous NIV initially until normalized blood pH ( > 7.35). We monitored EVT, Vmin, and leakage. We used BiPAP Synchrony and Autotrak (*Respironics Inc*.), and two types of facemasks: Mirage IV series mask (*Resmed*) and Series II full facemask *(Respironics*). We monitored EVT, Vmin, and leakage in order to program inspiratory pressure Levels and adjust the mask.

In addition to ventilatory support, both groups received bronchodilators, intravenous corticosteroids, and antibiotic therapy consisting of beta-lactam (piperacillin/tazobactan at 4.5 g IV every 6 hours) in combination with a new fluoroquinolone (Levofloxacin 500 mg IV daily).

Primary analysis: level of consciousness (Glasgow Coma Scale score). Secondary analysis: duration of mechanical ventilation, hospital stay, and progression (exhaled tidal volume, inspiratory pressure, and arterial blood gases).

### Statistical analysis

All data were expressed as mean ± standard deviation (SD) for continuous variables and as percentages for categorical variables. Continuous variables with normal distribution were examined using the Kolmogorov-Smirnov test, and were compared using Student's *t*-test. For categorical variables, χ^2^ or Fisher's exact tests were used as appropriate. We used analysis of variance (ANOVA) with repeated measures to compare the ability of different variables (pH, pCO_2_, HCO_3_, heart rate, respiratory rate, systolic blood pressure, diastolic blood pressure, EVT, Vmin, leaks, maximum programmed IPAP, and GCS) to predict the outcome of therapy in experimental and control patients. A *P* value <.05 was considered statistically significant.

## Results

**Figure 1:**
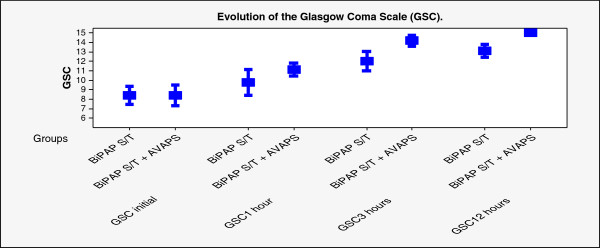
Describes the evolution of the glasgow coma score in both groups.

A total of 22 patients were analyzed: 11 in the control group (BiPAP S/T) and 11 in the experimental group (BiPAP S/T with AVAPS). The mean age of all patients was 78.68 ± 10.42 years, mean APACHE II score was 18.50 ± 2.56, 9 patients were women (40.9%) and 13 were men (59.1%). Four patients were diagnosed with COPD with pneumonia (18.2%) and 18 were diagnosed with infectious exacerbations of COPD (81.3%). Sixteen patients (73.8%) received NIV in the emergency and ICU. There were no statistically significant differences between the two groups in terms of BMI, age, APACHE II score, or initial GCS score (Table 1). One patient (4.5%) used a Respironics full face mask, and the other 21 patients (95.5%) used the Mirage IV series (Resmed). In patients undergoing NIV with BiPAP S/T and AVAPS, the programmed tidal volume on AVAPS was 622.73 ± 81.74 ml/kg (range: 500–700), with a programmed Vt/kg of 10.26 ± 2.23 ml (range 7.89- 11,83). The programmed maximum IPAP values (BiPAP S/T with AVAPS) were: 21.36 ± 3.04 cmH_2_O (initial), 20.82 ± 3.19 cmH_2_O (1 hour), 19.36 ± 3.80 cmH_2_O (3 hours), and 19.55 ± 3.45 cmH_2_O (12 hours). The ANOVA analysis revealed statistically significant differences in favor of AVAPS for pCO_2_*(P* = .03), respiratory rate (*P* = .01), maximum IPAP (*P* = .005), GCS score (*P* = .00001) (Figure 1), and EVT (*P* = .01) (Table 2). However, no significant differences were observed for length of stay (*P* = .15) or duration of NIV (*P* = .18) (Table 3).

**Table 1:** Initial patient assessment results

**NIV study groups (All 22 patients)**		**Mean**	**SD**	***P***
**BMI**	BiPAP S/T	26.22	2.87	.99
	BiPAP S/T +AVAPS	24.23	2.62	
**Age (years)**	BiPAP S/T	77.55	6.49	.10
	BiPAP S/T + AVAPS	79.82	13.53	
**APACHE II**	BiPAP S/T	18.45	2.50	.86
	BiPAP S/T + AVAPS	18.55	2.73	
**Initial GSC**	BiPAP S/T	8.36	1.43	1.00
	BiPAP S/T + AVAPS	8.36	1.63	
**Initial pH**	BiPAP S/T	7.28	0.02	.45
	BiPAP S/T + AVAPS	7.29	0.03	

*Statistically significant (*P* value <.05).

A total 22 patients. 11 patients of group BiPAP S/T and 11 patients of group BiPAP S/T +AVAPS.

**Table 2:** Evolution of blood gases, vital signs, and ventilatory parameters (mean ± SD)

**Variables**	**Groups**	**Initial**	**1 hour**	**3 hours**	**12 hours**	***P***
**GSC**	BiPAP S/T	8.3 ± 1.4	9.7 ± 2	12 ± 1.5	13 ± 1	.00001*
	BiPAP S/T + AVAPS	8.3 ± 1.6	11 ± 1	14.1 ± 0.8	15 ± 0	
**pH**	BiPAP S/T	7.28 ± 0.02	7.30 ± 0.05	7.31 ± 0.11	7.32 ± 0.12	.31
	BiPAP S/T + AVAPS	7.29 ± 0.03	7.34 ± 0.04	7.37 ± 0.11	7.37 ± 0.08	
**pCO**_**2**_	BiPAP S/T	64.8 ± 9.1	58.3 ± 8.7	53.2 ± 9	50.1 ± 6.5	.03*
	BiPAP S/T + AVAPS	63 ± 16.3	50.7 ± 11.2	45.4 ± 7.9	43.6 ± 6.5	
**PO**_**2**_	BiPAP S/T	66.6 ± 12.7	83.1 ±17.8	75.3 ± 26.7	79.7 ± 16.2	.31
	BiPAP S/T + AVAPS	71.5 ± 16.8	78 ± 19.1	87.5 ± 11.5	87.4 ± 18	
**HCO**_**3**_	BiPAP S/T	26.9 ± 5.7	24.4 ± 6.3	25.8 ± 4.6	27.1 ± 4.3	.19
	BiPAP S/T + AVAPS	24.4 ± 5	22.5 ± 3.5	23.7 ± 5.2	24.6 ± 4.3	
**Base excess**	BiPAP S/T	3.3 ± 6.9	0.1 ± 7	10.3 ± 31.7	3.6 ± 4.7	.06
	BiPAP S/T + AVAPS	−1.8 ± 5.7	2.8 ± 18	5.7 ± 19.8	2.9 ± 9	
**Systolic blood pressure**	BiPAP S/TS/T	125.1 ± 10	124.2 ± 12.6	130.4 ± 14.3	130.6 ± 13.8	.29
	BiPAP S/T + AVAPS	125.9 ± 17.3	131.1 ± 21.1	129.9 ± 18.4	123.5 ± 16.9	
**Diastolic blood pressure**	BiPAP S/T	73.9 ± 9.8	72.2 ± 8.4	71.8 ± 9.4	73.7 ± 10.7	.07
	BiPAP S/T + AVAPS	65.5 ± 11.6	69.8 ± 11.6	70.1 ± 11.1	65.9 ± 8.5	
**Heart rate**	BiPAP S/T	86.7 ± 9.1	82.1 ± 7.8	80.4 ± 5.8	79.1 ± 5.5	.07
	BiPAP S/T + AVAPS	82 ± 10.9	82.5 ± 9.9	72.8 ± 14.1	72. ± 11.2	
**Respiratory rate**	BiPAP S/T	27.9 ± 5.6	23.2 ± 3.5	21 ± 2.6	20 ± 1.61	.01*
	BiPAP S/T + AVAPS	29 ± 6.9	17.4 ± 3.2	18.5 ± 3.6	19.9 ± 5.1	
**Maximum delivered IPAP received**	BiPAP S/T	12.3 ± 0.9	12.6 ± 0.9	14.3 ± 0.8	14.7 ± 1	.005*
	BiPAP S/T + AVAPS	19.8 ± 2.2	18.3 ± 2.3	18 ± 2.6	17 ± 2.3	
**EPAP**	BiPAP S/T	5.9 ± 0.3	6 ± 0	6 ± 0	6 ± 0	.32
	BiPAP S/T + AVAPS	6 ± 0	6 ± 0	5.9 ± 0.3	5.9 ± 0.3	
**Minute volume**	BiPAP S/T	8.7 ± 3.1	9.2 ± 2.2	10.8 ± 1.4	10.6 ± 1.4	.17
	BiPAP S/T + AVAPS	8.5 ± 2.2	10.5 ± 2.5	11.5 ± 3.1	11.6 ± 1.8	
**Exhaled tidal volume**	BiPAP S/T	304 ± 60.6	400.5 ± 73.9	519 ± 61.4	531.1± 63.6	.01*
	BiPAP S/T + AVAPS	298.6 ± 54.3	606.3 ± 75.4	626.3 ± 77.6	617.6 ± 77.4	
**Leak**	BiPAP S/T	9.3 ± 3.8	21 ± 2	11 ± 3	11 ± 3.4	.20
	BiPAP S/T + AVAPS	14 ± 11.2	18.3 ± 3.7	17.5 ± 16	17.5 ± 16	

*Statistically significant (*P* value <.05).

^**^The ANOVA with repeated measures to compare the ability of different variables the both groups at 1, 3, 12 hours.

**Table 3:** Duration of hospital stay and time on NIV

	**Group**	**Mean**	**Standard deviation**	***P***
**Duration of hospital stay (days)**	BiPAP S/T	7.27	2.49	.15
	BiPAP S/T + AVAPS	7.09	1.45	
**Duration of NIV (days)**	BiPAP S/T	5.81	1.83	.18
	BiPAP S/T + AVAPS	5.36	1.12	

*Statistically significant (*P* value <.05).

## Discussion

Our study demonstrates that the addition of AVAPS to BiPAP S/T in patients with encephalopathy and infectious exacerbations of COPD produces a rapid recovery of consciousness (GCS), with early improvement of arterial blood gases as compared to conventional ventilation using solely BiPAP S/T. We observed significantly higher IPAP values in the BIPAP S/T + AVAPS group than in the group of patients treated solely with BIPAP S/T.

No studies exist in the medical literature describing the benefits of using NIV with AVAPS in acute patients. However, in chronic patients with obstructive sleep apnea and alveolar hypoventilation syndrome, authors report a rapid improvement in pCO_2_ and sleep quality using this technique [18-20]. Most studies reported in the literature describing successful use of NIV in hypercapnic encephalopathy indicate an improvement in GCS within only a few hours of initiating NIV, although the vast majority of these are clinical case reports or observational studies [6-8,21-26].

The goal in our study was, the rapid recovery of consciousness in a group of patients undergoing BIPAP S/T + AVAPS (target volume), we scheduled a target volume between (500-700ml) in our patients, with a Vt target average 10.26 ± 2.23 ml (range 7.89 to 11.83), with peak inspiratory pressures during therapy programmed to 26, once the patient achieved clinical stable condition and, in the target Vt our patients were reprogrammed to 6–8 ml/kg/ weight according to manufacturer's specifications. The decision was taken by the expert physician in charge of patient case-dependent (sensory severity scale or measured initial Glasgow).

BIPAP mode S/T + AVAPS delivered pressure changes progressively allowing the patient to conform much better to those pressures while the target tidal volume is reached.

Patients with the acute decompensation of COPD, accompanied by an altered mental status require rapid correction of alveolar hypoventilation which ensure an adequate tidal volume (minute volume) (volume settings between 8–12 ml/kg/weight) for rapid dissemination or carbon monoxide swept cerebrospinal fluid and brain and its sensory recovery as early as possible.

Studies examining the use of NIV in hypercapnic encephalopathy indicate that various ventilatory modes can be employed at different pressure levels. Gonzales et al [6] used BiPAP vision or BiPAP ST-D 30, in which IPAP levels were initially programmed at 12 cmH_2_O and increased every 4 hours with an IPAP value in the first hour of 17 + 2 cmH_2_O. In our study, patients on BiPAP S/T with AVAPS had an initial IPAP of 19.82, vs 12.36 in the control group. BiPAP S/T with AVAPS achieves the necessary inspiratory pressure level for a predetermined tidal volume, ensuring optimal pressure for the patient and facilitating a suitable inspiratory volume; this also rapidly overcomes alveolar hypoventilation, corrects pCO_2_ levels, and decreases CO_2_ levels in the brain so as to improve the patient's level of consciousness. Battisti et al. compared manually adjusted pressures with self-adjusting pressure support in patients with acute respiratory failure, which produced a decrease in pCO_2_ levels in the latter group [27].

Some studies found favorable results in patients using NIV in hypercapnic encephalopathy reduction in days of mechanical ventilation [26] reduced risk of nosocomial infection [28,29] and avoid intubation [30]. Recently, a pilot study tested the safety and efficacy of using an endotracheal tube through BiPAP in patients with a mean GCS of 6, a mean pH of 7.1, and poor management of Secretions, with a success rate of 85% (17/20) [31].

In our study, initial GCS and pH values were virtually equal between groups. Secretions were properly managed, which is essential for preventing technique failure and the need for endotracheal intubation. We observed a rapid and significant improvement in arterial blood gases and consciousness (GCS) in both groups; however, patients treated with BiPAP S/T + AVAPS improved much faster than patients treated with the conventional strategy, with a near-complete recovery within 3 hours. The improvement in the BiPAP S/T AVAPS group was probably linked to the rapid improvement in EVT and the fact that, in these patients, IPAP quickly reached the levels needed for maintaining appropriate tidal volume, and hypoventilation was corrected with consequent improvements in alveolar ventilation.

We observed no complications, gastric distention, or facial deformities, probably due to the fact that maximum system pressures did not surpass 20 cmH_2_O, which is the threshold associated with damage to the upper esophageal sphincter and facial structures [32,33].

Our study has certain limitations, including a small number of patients, despite the inclusion of three hospitals. In addition, the study was performed by a single research group with a long-term experience in NIV, which could create problems when extrapolating the results. Finally, it should be emphasized that our patients had a mean pH greater than 7.25, whereas other studies of hypercapnic encephalopathy have reported lower values. Other studies have also reported higher levels of pCO_2_ as causing altered levels of consciousness. The lower values of pCO_2_ that we observed could be due to several reasons. Firstly, some of our patients live at high altitude places. Gonzalez Garcia et al. [34], showed lower-than-normal pH and pCO_2_ values in patients with COPD undergoing effort or exercise [35]. Secondly, some of our patients might have had a very low pH with prolonged base excess and bicarbonate for patients with COPD. The effects of blood volume, diuretic use, height and affecting renal bicarbonate reabsorption have not been assessed [36]. We must also consider that patients in both groups experienced a rapid improvement in GCS of 2 points or more within 3 hours of starting treatment; a lack of improvement of 2 points could be a determining factor for rapid endotracheal intubation, which would obviously constitute an invasive procedure [34]. Finally, our study involved matched case-controls without randomization. Despite these limitations, we believe that this study provides valuable information, as it confirms the usefulness of NIV in hypercapnic encephalopathy, and upholds BiPAP S/T with AVAPS as a strategy that ensures safe and appropriate pressures and tidal volumes, facilitating a rapid correction of arterial blood gases, especially pCO_2_, and thus, minimizing the deleterious effects to the brain.

## Conclusions

We propose the use of BiPAP S/T with AVAPS as a safe strategy of noninvasive ventilatory treatment in patients with exacerbations of COPD and hypercapnic encephalopathy (GCS < 10), with the caveat that these patients should be treated in units with ample experience and under close surveillance.

## Abbreviations

APACHE II: Acute physiology and chronic health evaluation II; AVAPS: Average volume assured pressure support; BiPAP (S/T): Bilevel positive airway pressure (spontaneous/timed); EPAP: Expiratory positive airway pressure; EVT: Exhaled tidal volume; GCS: Glasgow Coma Scale; IPAP: Inspiratory positive airway pressure; NIV: Noninvasive mechanical ventilation; pCO2: Partial pressure of carbon dioxide; Vmin: Minute volume; Vt: Tidal volume; COPD: Chronic obstructive pulmonary disease.

## Competing interests

None of the authors have a competing of interests to declare in relation to this work.

## Authors’ contributions

KHBCl: contributed to the study design; data acquisition, analysis, and interpretation; drafting and revision of the manuscript; and preparation of the final version of the manuscript submitted. MHBC: contributed to the study design; analysis, and interpretation; and preparation of the final version of the manuscript submitted. MCS: contributed to the study design; data acquisition, analysis and revision of the manuscript. ANM: contributed to the study design; data acquisition and revision of the manuscript. RSE: contributed to the study design; data acquisition and revision of the manuscript. MM: drafting and revision of the manuscript preparation of the final version of the manuscript submitted. AER: preparation of the final version of the manuscript submitted. GGD: analysis and preparation of the final version of the manuscript submitted. MGA: drafting and revision of the manuscript; preparation of the final version of the manuscript submitted. All authors read and approved the final manuscript.

## Pre-publication history

The pre-publication history for this paper can be accessed here:

http://www.biomedcentral.com/1471-2466/13/12/prepub
